# Small RNA profiling in *Pinus pinaster* reveals the transcriptome of developing seeds and highlights differences between zygotic and somatic embryos

**DOI:** 10.1038/s41598-019-47789-y

**Published:** 2019-08-05

**Authors:** Andreia S. Rodrigues, Inês Chaves, Bruno Vasques Costa, Yao-Cheng Lin, Susana Lopes, Ana Milhinhos, Yves Van de Peer, Célia M. Miguel

**Affiliations:** 1 iBET, Instituto de Biologia Experimental e Tecnológica, Apartado 12, 2781-901 Oeiras, Portugal; 2 Instituto de Tecnologia Química e Biológica António Xavier, Universidade Nova de Lisboa (ITQB NOVA), Av. República, 2780-157 Oeiras, Portugal; 3 INESC-ID, Instituto Superior Técnico, Universidade de Lisboa, Rua Alves Redol 9, Lisboa, 1000-029 Portugal; 4 Biotechnology Center in Southern Taiwan and Agricultural Biotechnology Research Center, Academia Sinica, Tainan, Taiwan; 5 VIB-UGent Center for Plant Systems Biology, Ghent, Belgium; 6 Department of Plant Biotechnology and Bioinformatics, Ghent University, Ghent, Belgium; 7 Department of Biochemistry, Genetics and Microbiology, University of Pretoria, Private bag X20, Pretoria, 0028 South Africa; 8 BioISI – Biosystems & Integrative Sciences Institute, Faculdade de Ciências, Universidade de Lisboa, Lisboa, Portugal

**Keywords:** Plant embryogenesis, Small RNAs

## Abstract

Regulation of seed development by small non-coding RNAs (sRNAs) is an important mechanism controlling a crucial phase of the life cycle of seed plants. In this work, sRNAs from seed tissues (zygotic embryos and megagametophytes) and from somatic embryos of *Pinus pinaster* were analysed to identify putative regulators of seed/embryo development in conifers. In total, sixteen sRNA libraries covering several developmental stages were sequenced. We show that embryos and megagametophytes express a large population of 21-nt sRNAs and that substantial amounts of 24-nt sRNAs were also detected, especially in somatic embryos. A total of 215 conserved miRNAs, one third of which are conifer-specific, and 212 high-confidence novel miRNAs were annotated. MIR159, MIR171 and MIR394 families were found in embryos, but were greatly reduced in megagametophytes. Other families, like MIR397 and MIR408, predominated in somatic embryos and megagametophytes, suggesting their expression in somatic embryos is associated with *in vitro* conditions. Analysis of the predicted miRNA targets suggests that miRNA functions are relevant in several processes including transporter activity at the cotyledon-forming stage, and sulfur metabolism across several developmental stages. An important resource for studying conifer embryogenesis is made available here, which may also provide insightful clues for improving clonal propagation via somatic embryogenesis.

## Introduction

Zygotic embryogenesis is a crucial phase of the life cycle of seed plants, growth axes are established and accumulation of reserves takes place for later use during germination^1^. The study of zygotic embryogenesis has been challenging due to the small size of the embryo, particularly during early embryogenesis, and due its location deep within the seed tissues^2^.

Somatic embryogenesis, whereby a whole plant is derived from somatic cells that become competent and enter into the embryogenic pathway *in vitro*, is often the model system of choice to study the molecular regulation of plant embryogenesis^2^. Simultaneously, it has been used as a clonal propagation technology in several conifer species where somatic embryogenesis is induced mainly from immature and mature zygotic embryo (ZE) explants^3^. The study of the molecular regulation of zygotic embryogenesis is therefore very important in providing fundamental knowledge to improve the success of somatic embryogenesis protocols which are still largely empirical^4^.

Small non-coding RNAs (sRNAs) are important regulators of the expression of genes involved in plant development and in responses to both abiotic and biotic stresses^5^. The two main classes of sRNAs described in plants are microRNAs (miRNAs), derived from single stranded hairpin RNA precursors, and short interfering RNAs (siRNAs), derived from double stranded RNA precursors. Both classes of sRNAs are processed by DICER-LIKE (DCL) proteins. Plant miRNAs are typically 20–22-nt and act on targets through post-transcriptional gene silencing (PTGS), either by transcript cleavage or by translational repression. siRNAs may play their role by PTGS or transcriptional gene silencing (TGS) through alterations in DNA methylation. siRNAs are divided in different subclasses, the most common of which are the 21–22-nt secondary siRNAs, involved in PTGS and TGS, and the 24-nt heterochromatic siRNAs (het-siRNAs), involved in TGS^6^.

A survey of sRNA sequence and size profiles in 3 green algae and 31 vascular plant species revealed variability among plant species^7^. Overall, the 21-nt and 24-nt sequences prevail in the sRNA transcriptomes of angiosperms^7^, and during the seed development phase, diverse sRNAs size profiles seem to be present^8^. In the gymnosperm clade, including the conifers, contradictory results relating to the expression of 24-nt sRNAs have been reported^7,9–16^. The differences observed, as well as the distinct features of zygotic embryogenesis in angiosperms *versus* gymnosperms, show that *Arabidopsis thaliana* is not the best model for studies on somatic embryogenesis in non-angiosperm species.

The miRNAs are by far the better characterized class of sRNAs. In v.22 of miRBase^17^, a total of 8485 plant miRNAs have been deposited, however only 669 have been identified in conifer species. Compared with angiosperms, there is little information on sRNAs available for conifers. Nonetheless, many miRNAs appear to be specific for conifers.

Reports on high-throughput small RNA sequencing (RNA-Seq) in conifers have covered pools of different tissues, including seeds from *Cunninghamia lanceolata*^14^, seeds from *Picea glauca*^18,19^, needles from *Pinus contorta*^11^, over 20 different tissues from *Picea abies*, including male and female cones^7,13,20^, male and female cones from *Pinus tabuliformis*^12^, and different pooled tissues from *Larix leptolepis*, including somatic embryos (SEs), or seedling tissues^15,16^.

The current knowledge on sRNAs expression and function in seed development has been recently reviewed^8^, pointing to a very limited number of miRNAs whose function during this phase of the plant life cycle has been experimentally characterized (namely miR156, miR159, miR160, miR164, miR165/166, miR172, miR394, and miR397).

Indirect evidence of the relevance of sRNAs throughout *Pinus pinaster* (maritime pine) embryogenesis has been highlighted in a genome-wide transcriptomic analysis of consecutive stages of embryo development^21^. This study revealed that sRNA-associated functions such as *siRNA and miRNA binding*, and *gene silencing by miRNA*, are differentially regulated across *P. pinaster* zygotic embryogenesis. Also, miRNA functions appeared more represented in mid to late embryogenesis^21^. In addition to conserved miRNAs that are expected to be involved in conifer and angiosperm embryogenesis, we hypothesize that the function of conifer-specific sRNAs may underlie some of the characteristic differences between the developing embryos of conifers *versus* those of angiosperm species. In this work, we provide an overview of the sRNA transcriptome of *P. pinaster*, an important conifer species widely spread throughout the Mediterranean region where it has a significant economic relevance. A set of miRNAs, including conserved and newly discovered miRNAs, have been identified, some of which show evident differences in their expression throughout embryo development. This dataset represents an important resource for the future characterization of the functional roles of sRNAs in conifer embryogenesis and advances the current knowledge of the molecular regulation of this process. Finally these results provide clues for how to improve clonal propagation via somatic embryogenesis.

## Results

### Small RNA-Seq data

**Figure 1:**
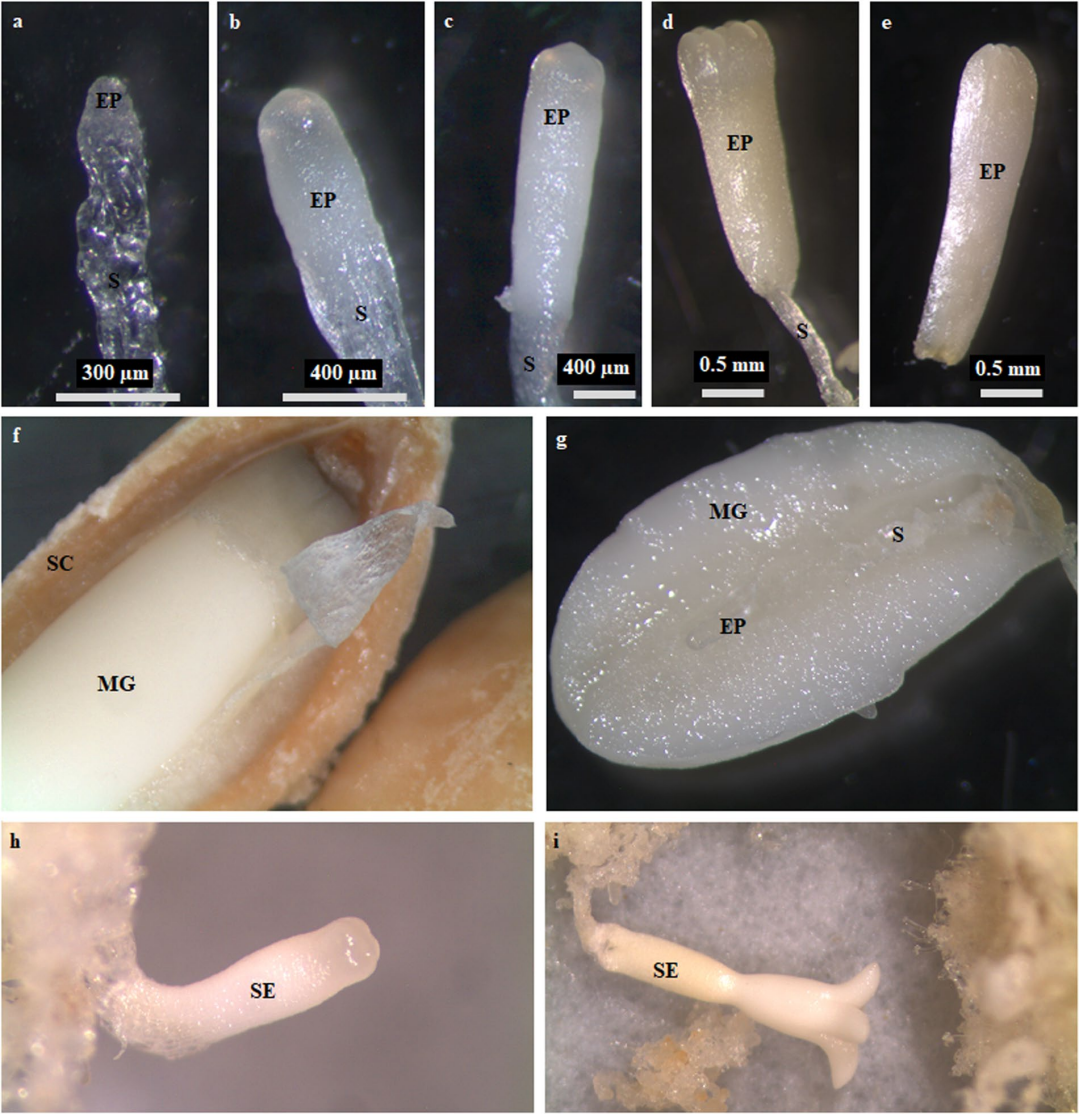
Biological material used to prepare the sRNA libraries. (**a**–**e**) Developmental stages representative of the zygotic embryo samples, namely (**a**) ZE0, (**b**) ZE3, (**c**) ZE4B, (**d**) ZE5 and (**e**) ZE7. (**f**) Open seed with exposed megagametophyte tissue. (**g**) Open megagametophyte with exposed embryo. Somatic embryos at the developmental stages (**h**) SE4B and (**i**) SE7. The legend is: embryo proper (EP), suspensor (S), seed coat (SC), megagametophyte (MG) and somatic embryo (SE).

In order to obtain a comprehensive, dynamic sRNA transcriptome of the *P. pinaster* seed throughout embryo development, sRNA libraries of ZEs at five stages of development from early to late embryogenesis (ZE0, ZE3, ZE4B, ZE5, ZE7), and corresponding megagametophytes (MGs) at three stages of development (MG0, MG4B, MG7), were sequenced using Illumina technology. In addition, sRNA libraries from SEs were also sequenced to characterize putative differences between zygotic and somatic embryogenesis during mid and late embryogenesis (see Fig. 1). Small RNA-Seq data was obtained from 16 libraries (Supplementary Table S1A–C), yielding between 13,295,829 and 33,047,491 raw reads per library and almost 330 M raw reads in total. After filtering (to remove low complexity and t/rRNA sequences) about 110 M reads of 18-nt to 26-nt and an absolute abundance of > = 5 were retained. These sequences represent 19–41% of the initial raw reads in each sRNA library. From these, approximately 76 M had a perfect match to the genome of *Pinus taeda*, which corresponds to about 808 K unique (distinct) reads. The percentages of filtered reads and of reads aligned to the genome were consistent between biological replicates.

**Figure 2:**
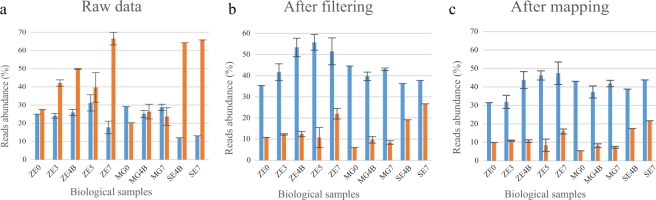
Distribution of 21-nt (blue columns) and 24-nt (orange columns) reads across several stages of development of zygotic and somatic embryos (ZEs and SEs respectively) and megagametophytes (MGs) in the (**a**) raw sequence data, (**b**) after filtering and (**c**) mapping against *Pinus taeda* genome. In the x-axis are represented the biological samples, including ZEs (ZE0-ZE7), MGs (MG0-MG7) and SEs (SE4B-SE7). The y-axis represents reads abundance displayed as percentage values relative to the total reads considered at each step. In cases where biological replicates were available (ZE3, ZE4B, ZE5, ZE7, MG4B, and MG7), the columns represent the mean value and the *range error bars* encompass the minimum and maximum values.

The size profiles of reads expressed per biological sample were analysed (Fig. 2) focusing on 21-nt and 24-nt reads. After filtering and mapping, the 21-nt reads are clearly more highly expressed in all samples, but the 24-nt reads are also clearly present and are more represented in the later stages of embryogenesis (from embryo stages ZE4B and SE4B onwards).

### Annotation of the miRNA transcriptome of *P. pinaster* embryogenesis

Genome-aligned reads were initially mapped against mature miRNAs deposited in miRBase v.21 to annotate conserved miRNAs. Then, both the annotated conserved miRNAs and the remaining unidentified sRNAs were further analysed to: (1) predict putative novel miRNAs and (2) identify miRNA precursor and miRNA-star (sequence complementary to the mature miRNA in the single stranded hairpin RNA precursor) sequences for annotated miRNAs. Furthermore, miRNA-star sequences were also annotated. Identified conserved, novel and star miRNAs can be found in Supplementary Table S2A–C.

**Figure 3:**
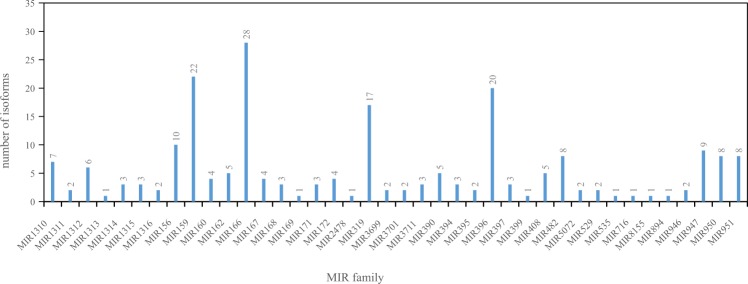
Absolute number of isoforms in each conserved miRNA family annotated in the sRNA libraries. The reads that perfectly mapped in the genome of *Pinus taeda* were compared with mature miRNAs deposited in miRBase v.21, and those aligned with up to two mismatches were annotated as an isoform of the conserved miRNA deposited in miRBase v.21. At the top of each column is the exact number of isoforms.

A total of 215 conserved miRNAs were annotated, corresponding to 40 conserved miRNA families (MIRs), each containing between 1 and 28 isoforms (Fig. 3). The conserved miRNA families with the highest number of isoforms were MIR166 (28), MIR159 (22), MIR396 (20) and MIR319 (17). Many of the conserved miRNA families were present across all of the libraries, namely MIR1311, MIR1312, MIR156, MIR162, MIR166, MIR167, MIR168, MIR172, MIR319, MIR3711, MIR390, MIR396, MIR482, MIR946, MIR947 and MIR951. A total of 8266 novel miRNAs were predicted of which 212 have a miRNA-star sequence and are thus considered as high-confidence *P. pinaster* novel miRNAs (see Supplementary Table S2A–C).

The first 5′-end nucleotide of the sRNA may indicate preferential association to a specific ARGONAUTE (AGO) protein^22^. In our study, a total of 4042 (46%) mature miRNAs begin with nucleotide A, 3713 (42%) with U, 530 (6%) with C and 472 (5%) with G. Conserved miRNAs were predominantly found with a 5′-end U, whereas the majority of novel miRNAs displayed a 5′-end A or a 5′-end U. This suggests that most *P. pinaster* miRNAs play their regulatory roles upon being selectively loaded by AGO1 (5′-end U miRNAs) or AGO2 (5′-end A miRNAs)^23^.

**Figure 4:**
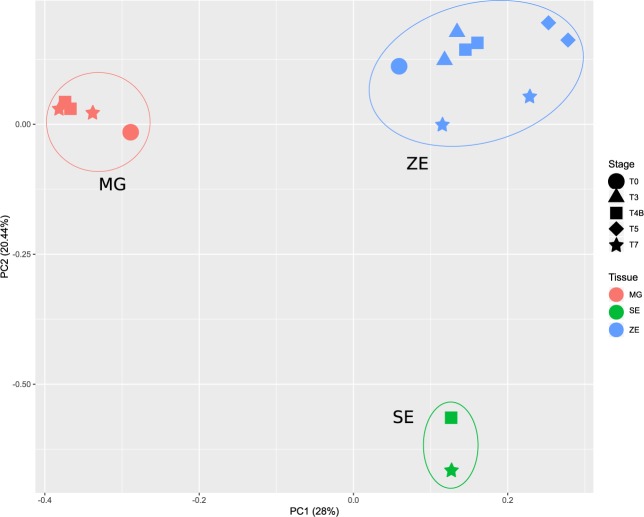
Principal Component Analysis of all sRNA libraries using the transformed expression values, ln(counts per million, CPM), for each conserved or novel miRNA. In the biplot of first two principal components (PCs), PC1 and PC2 account for 28% and 20.44% of variation in the data, respectively. PC1 translates the influence of miRNAs whose expression values is most distinct between embryos and megagametophytes, whereas PC2 translates the influence of miRNAs whose expression values is most distinct between zygotic embryo (ZE) and somatic embryo (SE) samples. The legend for developmental stages (geometric shapes) and tissues (colours) is: ZE0 (blue circle), ZE3 (blue triangle), ZE4B (blue square), ZE5 (blue diamond), ZE7 (blue star), MG0 (pink circle), MG4B (pink square), MG7 (pink star), SE4B (green square), SE7 (green star).

A principal component analysis (PCA) of all libraries, using conserved and novel miRNAs, demonstrates that the three types of tissues can be distinguished based on their miRNA profiles (Fig. 4). Approximately 48% of the variance can be explained by the two principal components. Distinct developmental stage samples from the same tissue are highly correlated and therefore cluster together. It should be noted that hierarchical cluster analysis (HCL) showed consistency between biological replicates (Supplementary Fig. S1).

**Figure 5:**
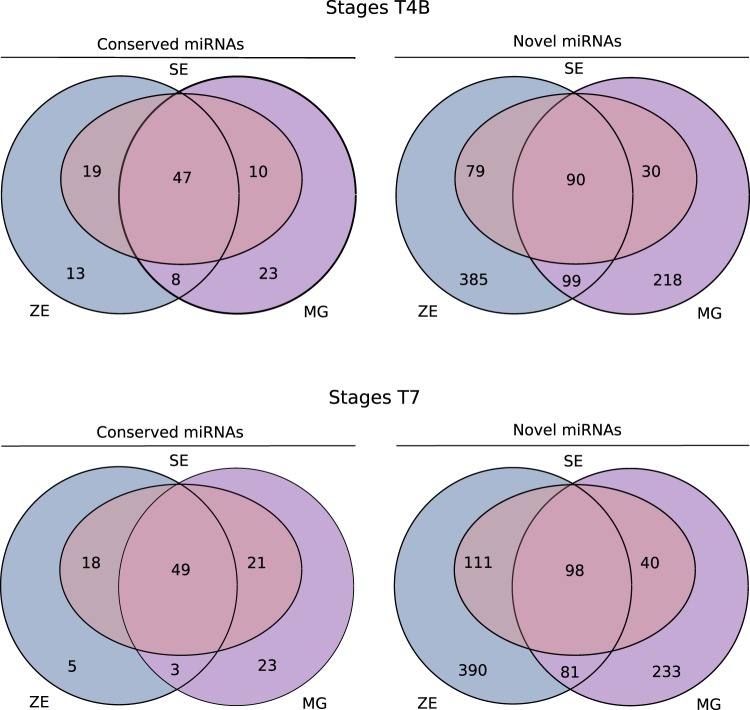
Venn diagrams of the total conserved and novel miRNAs found in zygotic embryos (ZEs), somatic embryos (SEs) and megagametophytes (MGs) at stage T4B (top panel) and T7 (bottom panel). Only miRNAs which have been annotated in both biological replicates were considered in this comparison, thereby excluding those unique to SEs.

A comparison of the miRNA transcriptomes of ZEs, SEs and MGs at stages T4B and T7 showed a very similar number of total annotated miRNAs in stages T4B and T7 (Fig. 5) with 1071 miRNAs and 1098 miRNAs, respectively. Only miRNAs which have been annotated in both biological replicates were considered in this comparison, thereby excluding those unique to SEs. The number of conserved miRNAs annotated in each type of tissue, either at stage T4B or stage T7, is very similar and most conserved miRNAs are detected in all of the three tissues. On the contrary, many annotated novel miRNAs seem to be present in only one of the tissues, with 653–680 novel miRNAs annotated in ZEs, 437–452 in MGs and 199–249 in SEs. The total number of miRNAs simultaneously present in ZEs and MGs decreased from stage T4B to stage T7, while miRNAs detected in both SEs and MGs increased.

### Overall expression patterns of conserved and novel miRNAs

**Figure 6:**
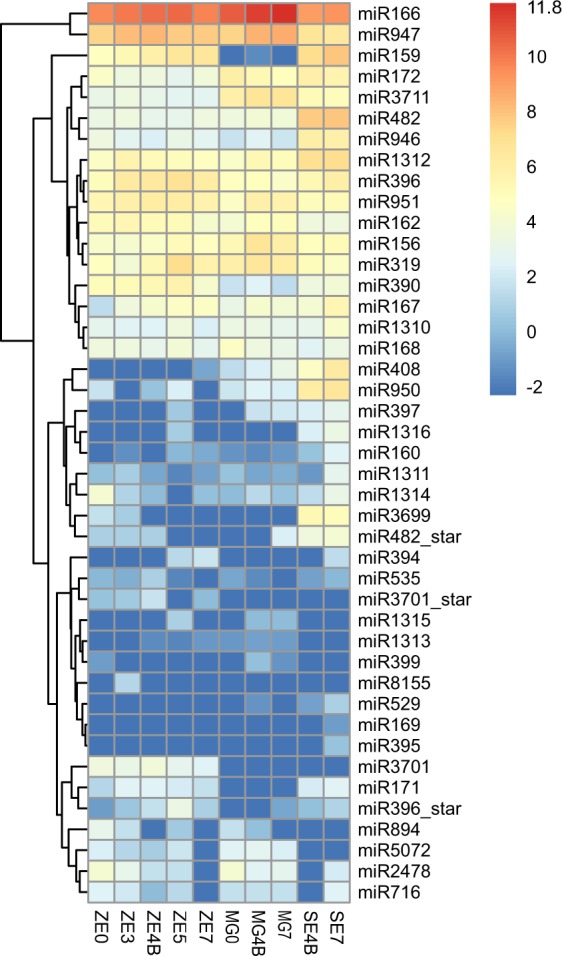
Conserved miRNA families detected across the distinct developmental stages within each tissue (ZE, MG and SE). The heatmap represents the transformed expression values for each miRNA family, and miRNAs are grouped according to expression profiles across the biological samples.

An overall analysis of the expression patterns of conserved miRNA families across all samples demonstrates that, with the exception of MIR166 and MIR947, these families are expressed at low to moderate levels in all libraries (Fig. 6). Note however that large variations in the expression levels of different isoforms within the same miRNA family across development might be found (Supplementary Fig. S2).

Given that SE development closely resembles zygotic embryogenesis but it occurs *in vitro* without a surrounding MG, it was interesting to note that some miRNA families, such as MIR397 and MIR408, were moderately expressed in SEs and MGs but were greatly reduced in ZEs. Other families, such as MIR394, MIR159 and MIR171, exhibited moderate expression in embryos (ZEs and SEs) but were greatly reduced in MGs (Fig. 6). The majority of the novel miRNAs also exhibited low to moderate expression levels, and similarly to conserved miRNAs, there are several examples of tissue specific expression (Supplementary Table S2A–C).

In order to highlight those miRNAs that are highly expressed during embryo development, a cut-off of 100 counts per million (CPM) was applied to miRNAs expressed either by ZEs or MGs, at stages T4B and T7. A shortlist containing 20 conserved miRNAs and 21 novel miRNAs (Supplementary Table S2A–C) that passed this criterium, was generated. Within this list, four conserved miRNAs were simultaneously present in ZEs and SEs at stages T4B and T7 (Fig. 5), namely miR166-TCTCGGACCAGGCTTCATTCC, miR159-TTTGGTTTGAAGGGAGCTCT, miR159-TTTGGTTTGAAGGGAGCTCTA and miR159-TTGGATTGAAGGGAGCTCCA. The shortlist also includes miR166-TCGGACCAGGCTTCATTCC and miR319-CTTGGACTGAAGGGAGCTCCC, simultaneously present in MGs and SEs at stages T4B and T7 (Fig. 5). miR162-TCGATAAACCTCTGCATCCGG which is described as targeting *DCL* transcripts^24^, is less expressed in SEs. Eleven of the novel miRNAs in this list are high-confidence, and their expression is generally lower in SEs than in ZEs or MGs.

### Differentially expressed miRNAs in zygotic embryos and megagametophytes

**Figure 7:**
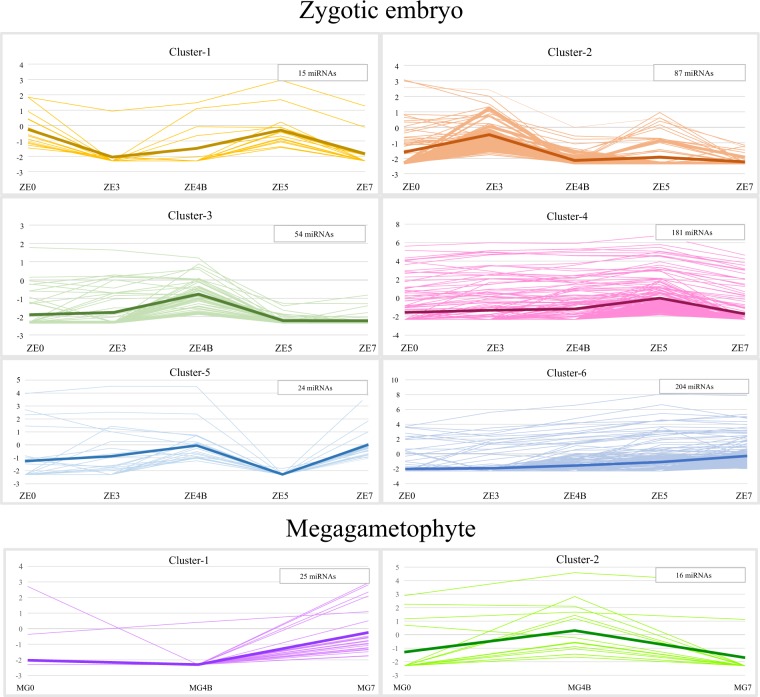
Clustering of the 565 DE miRNAs detected in ZE libraries (top six clusters) and the 41 DE miRNAs detected in MG libraries (bottom two clusters), according to their expression profiles. The y-axis represents expression values as ln(CPM). In each cluster the thicker line represents the mean expression value, and the insert shows the number of miRNAs represented.

A total of 565 differentially expressed (DE) miRNAs were detected among the 1710 miRNAs present across *P. pinaster* ZE development, including 36 conserved miRNA isoforms from 17 miRNA families, 504 novel miRNAs and 25 miRNA-stars. The DE miRNAs in ZE samples were grouped into six distinct clusters according to their expression profiles (Fig. 7; Supplementary Table S3A–F).

The expression profiles can be associated with a specific ZE stage(s): cluster-1 (15 miRNAs) presents two peaks of expression, at ZE0 and ZE5; cluster-2 (87 miRNAs) shows a single expression peak at ZE3; cluster-3 (54 miRNAs) has a peak at ZE4B; cluster-4 (181 miRNAs) a peak at ZE5; cluster-5 (24 miRNAs) has two peaks of expression, at ZE4B and ZE7; and in cluster-6 (204 miRNAs) the expression steadily increases up to ZE7. These results show that the majority of DE miRNAs throughout ZE development reach their peak of expression late in embryogenesis, at the cotyledonary (ZE5) and mature (ZE7) embryo stages.

Another 41 miRNAs were DE between MG4B and MG7 samples, where 25 are up-regulated at MG7 (cluster-1) and 16 miRNAs are up-regulated at MG4B (cluster-2), thereby grouping into two distinct expression profiles (Fig. 7; Supplementary Table S4A,B). Among the 41 DE miRNAs are four miRNA isoforms from four conserved miRNA families (MIR319, MIR396, MIR482 and MIR950) and 37 novel miRNAs. Similarly to the ZE samples, the largest number of DE miRNAs in MGs appear to reach their peak of expression late in embryogenesis.

### Gene enrichment analysis of predicted miRNA targets

To link miRNA differential expression patterns with putative functions in ZE and MG development, their target transcripts were predicted against the *P. pinaster* transcriptome (Supplementary Table S5A–F and Supplementary Table S6A,B), and associated GO terms were retrieved for each expression profile cluster (Supplementary Table S7).

In the ZE samples, the enrichment analysis of the predicted targets retrieved a total of 167 overrepresented Gene Ontology (GO) terms, namely 92 Biological Process (BP), 55 Molecular Function (MF), and 20 Cellular Component (CC) terms. The targets of miRNAs in clusters 1 and 5 are mostly associated with *sulfur compound metabolism* and *sulfur compound biosynthesis*. In cluster 2, most miRNA target genes are related to *aspartate family amino acid metabolism*, including *organic acid metabolism* and *carboxylic acid metabolism*, both also found overrepresented in cluster 4 (Supplementary Table S7 and Supplementary Fig. S3). Interestingly, the majority of the targets of cluster 3 miRNAs are associated with *transporter activity*. An overrepresentation of targets related with *branched-chain amino acid biosynthesis*, *cell communication* and *signaling* is exclusive to miRNAs in cluster 4, while *regulation of phosphate metabolism*, *biological regulation* and *phosphorus metabolism* are overrepresented in the targets of miRNAs in clusters 4 and 6 (Supplementary Table S7, Supplementary Fig. S4 and Supplementary Fig. S5). It should be noted that the highest number of enriched BP terms were associated with the miRNA targets of cluster 6, including *cellular metabolism*, *cellular process*, *protein phosphorylation*, *cellular localization*, *cellular carbohydrate metabolism* or *cell cycle* (Supplementary Fig. S5).

The enrichment analysis of the predicted targets in the MG samples retrieved a total of 21 overrepresented GO terms (ten BP, nine MF, and two CC). The results show an overrepresentation of targets associated with the MF term *protein binding* in both clusters 1 and 2. Cluster 2 contains several overrepresented BP terms such as *response to stimulus*, *response to organic substance*, *cell communication*, *signaling*, *nucleocytoplasmic transport* and *glucose metabolism* (Supplementary Table S7 and Supplementary Fig. S6).

### MiRNAs expression validation by RT-qPCR

**Figure 8:**
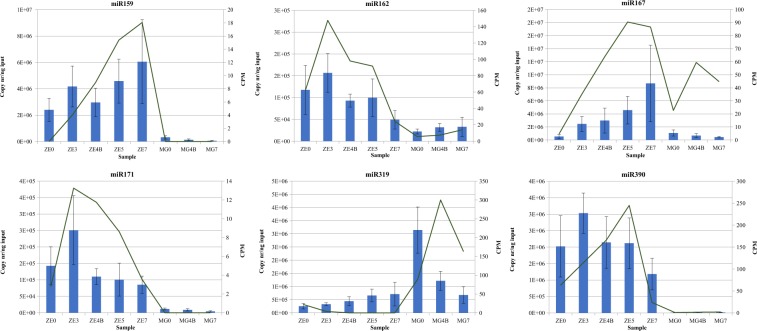
Differentially expressed conserved miRNAs validated with RT-qPCR in ZE and MG samples. RT-qPCR values (copy number/ng input) are represented as columns and RNA-Seq values (CPM) as continuous lines. *Error bars* were calculated for the RT-qPCR values, from at least two biological replicates.

Six conserved miRNAs that were highly and/or differentially expressed in ZE development, were selected for RT-qPCR validation (Fig. 8). The sequences of the TaqMan miRNA probes selected for RT-qPCR validation fully matched the miRNA sequences found in the DE analysis. The RT-qPCR results showed a good agreement with expression profiles from small RNA-Seq data. All validated miRNAs, with the exception of miR319, had higher expression in ZEs than in MGs.

## Discussion

We have surveyed the sRNA transcriptome of the developing ZE, SE and MG of *P. pinaster*. Only four conifer species have their miRNAs deposited in miRBase, the reference miRNA database, namely *Cunninghamia lanceolata*, *Picea abies*, *Pinus densata* and *Pinus taeda* (http://www.mirbase.org/cgi-bin/browse.pl, accessed on 08-June-2018). For *Picea abies*, the first conifer with a publicly available reference genome^13^, 594 miRNA precursors and 600 mature miRNAs are deposited, including many distinct isoforms from the same miRNAs family. Here, we have identified and annotated a total of 215 conserved miRNAs and 212 high-confidence putative novel miRNAs in embryos and in the MG of *P. pinaster*, which represent an important resource for the study of embryogenesis in conifer species and significantly enriches the current repertoire of published and annotated plant miRNAs.

To our knowledge, this is the most complete report disclosing the sRNA transcriptome of seed tissues of a conifer species across a developmental time course (obtained through next generation sequencing). Both 21-nt and 24-nt sRNAs increase towards the later stages of development, peaking at embryo maturation (ZE7). The considerable and diverse fraction of 24-nt sequences expressed by the analysed *P. pinaster* tissues consists of many unique sequences expressed at low levels. Therefore, the 21-nt sRNAs become the predominant fraction in the sRNA population as a result of the strong reduction of the 24-nt sRNAs after filtering out sequences with an absolute abundance lower than five. It should also be noted that the 24-nt reads are more abundant in ZEs and SEs than they are in MGs. Since the work published by Dolgosheina *et al*.^9^, the ability of conifers to express significant numbers of 24-nt sRNAs has been a topic of debate. The major plant sRNA classes which include 24-nt sequences, are the hairpin-derived siRNAs (hp-siRNAs), the natural antisense siRNAs (natsiRNAs), and the het-siRNAs^6^. Het-siRNAs are considered the most abundant siRNAs expressed by plants and, unlike hp-siRNAs and natsiRNAs, are solely composed of 24-nt sequences and associated with TGS via RNA-directed DNA methylation (RdDM). Het-siRNAs seem to be particularly important during plant meiosis, gametogenesis and embryogenesis, playing a role in the silencing of transposons and repetitive sequences in order to maintain genome integrity^6^.

In *Larix leptolepis*, a significant fraction of non-redundant 24-nt reads was also detected in somatic embryogenesis tissues, whereas non-redundant 21-nt sRNAs predominated in seedlings^15,16,25^. Similarly to our results, the 24-nt sRNAs exhibited lower redundancy, i.e. many diverse 24-nt sRNAs with low individual expression levels^16^. In *Cunninghamia lanceolata*, a single sRNA library prepared from seeds, calli, seedlings, adult leaves and stems, exhibited a higher abundance of the 24-nt sRNAs in both unique and redundant sRNAs size profiles^14^. In *Picea glauca*, 24-nt sRNAs were amongst the most abundant found at seed set^19^, although a decrease in expression of 24-nt sRNAs was observed as seed set progressed into maturation^18^. Moreover, there were more unique 24-nt sRNAs than 21-nt sRNAs, which was particularly evident at early stages of seed set^18^. Significant levels of 24-nt sRNAs were also found in reproductive tissues of *Picea abies*, in particular in male cones, which were found to be largely associated with repeats^13^. In *Pinus tabuliformis* immature male and female cones, both total and unique sRNA length distributions peaked at 21-nt, which together with the expression of sRNA biogenesis associated genes, pointed to the predominance of the miRNA pathway in these tissues^12^.

While in *P. pinaster* embryos the abundance of total 24-nt sRNAs increases towards maturation (ZE7 and SE7), being even higher in SEs than in ZEs, the opposite was observed during seed development of angiosperms such as canola, barley, rice and wheat^8^. Interestingly, a previous study reported overrepresentation of putative *AGO9* transcripts in the coding transcriptome of late *P. pinaster* embryos^21^. AGO9 belongs to a phylogenetic clade (AGO4/6/8/9) known to preferentially associate with 24-nt siRNAs and acting to silence transposons and repetitive sequences at the transcriptional level^22^. In *Picea abies*, a correlation was established between a much higher level of 24-nt siRNAs and increased CHH DNA methylation in somatic embryogenesis culture cells when compared with needles, which showed mostly 21-nt siRNAs^26^. Although no functional evidence is available, a putative role in epigenetic silencing of transposons and repetitive elements seems likely, which would be in agreement with the more frequent epigenetic/genetic instability often associated with *in vitro* culture^27^. However, it should be noted that although genetic and epigenetic instability has been detected in conifer embryogenic cultures, especially when these are maintained *in vitro* during long periods of time^26,28,29^, this risk has been considered limited when compared to angiosperm species^30,31^. This further suggests that 24-nt sRNAs might have a particularly relevant role in maintaining such stability in conifers.

It is now clear that conifer species are able to express considerable levels of 24-nt sRNAs but it remains to be clarified whether their expression dynamics are directly associated with the species, the stage of the life cycle and/or the type of tissue. The case for conifers might be similar to that of the *Selaginella* lineage, in which the het-siRNAs pathway was found to be active only in specific tissues^32^.

We have annotated a high number of novel and conserved miRNAs from *P. pinaster* embryos and MGs. Many miRNA families detected here are conserved across land plants, such as MIR156, MIR159, MIR160, MIR162, MIR166, MIR167, MIR168, MIR169, MIR171, MIR172, MIR319, MIR390, MIR394, MIR396, MIR397, MIR529 and MIR535. Other families such as MIR482, which was found to be poorly enriched in monocots, or MIR395, MIR399 and MIR408, which are enriched in angiosperms^7^, are also present in *P. pinaster* embryos and MGs. Thirteen miRNA families are conifer-specific (MIR946, MIR947, MIR950, MIR951, MIR1311, MIR1312, MIR1313, MIR1314, MIR1315, MIR1316, MIR3699, MIR3701 and MIR3711).

It has been documented that the more conserved the miRNA sequence is, the more abundant it is^7^. In this work, miR166-TCGGACCAGGCTTCATTCCCC with 21-nt is the most abundant sequence across all libraries but there are also several highly abundant putative novel miRNAs such as novel-TCCAACGAAGATCAGAAGGCTT with 22-nt. However, in general, the average expression of conserved miRNAs is higher than that of novel miRNAs.

The analysis of DE conserved and novel miRNAs suggests that the biological functions of miRNAs are particularly important in late *P. pinaster* embryogenesis. This supports the importance of posttranscriptional regulation by miRNAs during seed maturation. Furthermore, it is in close agreement with previous work highlighting that sRNA associated processes are differentially expressed during *P. pinaster* zygotic embryogenesis and provides further evidence of miRNA functions in mid to late embryogenesis^21^.

The function of a few conserved miRNAs annotated here had already been experimentally validated in seed development^8^. MIR159, MIR171 and MIR394 were found to be present in both ZEs and SEs, but greatly reduced in MGs. MIR159 is one of the largest miRNA families present in our data. Its increasing expression across embryogenesis, with several DE miR159 isoforms, points to an important function during late embryogenesis (ZE5 and ZE7). MiR159 has been identified as having a possible involvement in the plastid fatty acid biosynthesis pathway during seed maturation in *Brassica napus* and, a negative correlation between the expression of miR159 and its predicted target *KASII* (also known as *FAB1*) was confirmed^33^. Also in *P. pinaster* embryos, we found a homolog of *KASII* (sp_v3.0_unigene24638), encoding “3-ketoacyl-ACP synthase II”, as a predicted target of miR159. A *P. pinaster* homolog of *MYB101*, sp_v3.0_unigene11630, was also predicted as target of the DE isoforms of miR159, expression of which increase towards embryo maturation. These results are consistent with previous work reporting that *Arabidopsis* miR159 cleaves *MYB33* and *MYB101* transcripts during seed germination, contributing to block ABA signaling associated with seed dormancy^34^. In the conifer *Larix leptolepsis LaMYB33* was confirmed as target of miR159 in SEs. A negative correlation was observed between expression of *LaMYB33* and the expression of miR159 during the late stages of SE maturation^35^.

Both MIR159 and MIR171 have been pointed out as potential markers of embryogenecity after somatic embryogenesis induction in conifers^4^. The GRAS-family transcription factors *SCARECROW-LIKE* (*SCL*) are well known miR171 targets, although functional diversification was already described for some members of the MIR171 that are predicted to target non-*SCL6* genes^36,37^. MIR171 is a promising candidate to understand initial embryo-specific molecular processes and their relevance in zygotic *versus* somatic embryogenesis since, like MIR159, it is also exclusively expressed by ZEs and SEs of *P. pinaster*. In *Citrus sinensis*, miR171 could be detected in embryogenic calli but not in non-embryogenic calli. Based on its *SCL* targets, it was suggested that miR171 inactivates postembryonic growth to maintain normal somatic embryogenesis^38^. Amongst the predicted targets of the DE miR171 isoform in *P. pinaster* are the C-terminus sequence of a putative *SCL* and several genes encoding Mitogen activated protein kinase (MAP kinase, MPK).

MIR394, detected only during the late stages of *P. pinaster* embryo development (ZE5, ZE7 and SE7), has been detected as early as the 16-cell stage and up to the torpedo stage in wild-type *Arabidopsis* embryogenesis^39^. In *Arabidopsis*, miR394 repression of the *LEAF CURLING RESPONSIVENESS* (*LCR*) transcript was shown to be necessary to maintain the stem cell competence of the shoot apical meristem^39^. Finally, in *Brassica napus* the repression of *LCR* by miR394 was shown to play a role in seed development, where it impacts the seed content of storage oil, protein and glucosinolates^40^.

Due in part to the difficulties of isolating ZEs, especially at very early stages of development, somatic embryogenesis has been used as a model system for studying ZE development. However, somatic embryogenesis in conifers is usually artificially induced in the presence of high levels of auxin, which can lead to altered expression of many genes. From this work it is clear that SEs have different sRNA profiles when compared to their zygotic counterparts, although they express roughly the same repertoire of conserved and novel miRNAs. The miR482/miR2118 superfamily, found to be up-regulated in *P. pinaster* SEs, has been recently characterized in *Picea abies*. It was suggested that the miR482/miR2118 superfamily has dual functions in gymnosperms, regulating not only the production of phased secondary siRNAs from *NB-LRR* genes, but also triggering siRNA production in reproductive tissues^41^. Interestingly, these functions have been divergently retained in eudicots and monocots^42–45^. We do not know what roles this miRNA family is playing in SEs, but it may be involved in triggering siRNA production, which would be consistent with the higher amounts of 24-nt sRNAs observed for SEs when comparing to ZEs. On the other hand, the reported involvement of *NB-LRR* genes in hormonal responses to environmental stress^46^ would be in agreement with the stress inducing conditions used *in vitro* for SEs.

Other miRNA families present in SEs and MGs but greatly reduced in ZEs, are MIR397 and MIR408. Several studies on miR397 have reported roles in the abiotic stress response^47^ and MIR408 plays important roles during vegetative development in *Arabidopsis*^48–50^. The function of MIR408 in reproductive development remains unclear. MIR408 targets transcripts encoding copper-containing proteins and sucrose was shown to be an important regulator of such proteins through miR408 and miR398, which are induced by SPL7 in response to high sucrose^51,52^. Overexpression of MIR408 in *Arabidopsis* also altered various morphological traits including flower size and silique length, resulting in enhanced biomass and seed yield^53^. In maize endosperm the expression of miR408 was reported to be significantly altered following treatment with sucrose^54^.

Development of SEs can occur under high concentrations of sucrose in several conifer species, including *P. pinaster*. Moreover, the artificial conditions that are imposed can be considered stressful for the plant cells. In this context, and according to previous reports, it seems likely that the higher levels found for miR397 and miR408 in SEs are related, at least partially, to such conditions. The complete absence of a MG tissue in somatic embryogenesis is also a distinctive characteristic relative to zygotic embryogenesis, and we may speculate that some of the overrepresented functions in SEs are balancing the lack of a MG tissue. MIR3711 is one of the few miRNA families up-regulated only in MGs. This family of miRNAs has been also detected in *Picea abies* SEs but the target genes and putative functions are unknown^55^.

Regarding the putative novel miRNAs identified here, there are several interesting candidates for further characterization, for instance novel-TGAGATTGTTGGAGAGGTTCA and novel-AATGGGTTGACTGGAAAGACC (Supplementary Table S2A–C) expressed by both ZEs and SEs, but expressed lowly or entirely absent in MGs. Although these two examples refer to high-confidence novel miRNAs, the high number of predicted targets for most of them makes it difficult to hypothesize about their roles during embryogenesis, and functional studies are required.

Overall, from the cluster analysis of DE miRNAs, each stage of development seems to be associated with regulation by a characteristic population of miRNAs, which peak at defined time points. Gene enrichment analysis of the predicted targets of DE miRNAs, both conserved and novel, revealed enriched GO terms associated to different expression profiles. For instance, in stage T4B, transporter activity is highlighted, possibly in relation to the appearance of the cotyledons which requires a very active transport of auxin and nutrients to the developing tissues. Sulfur metabolism also featured in relation to DE miRNAs with varying expression profiles (clusters 1 and 5). Developing seeds are an important sink for oxidized or reduced sulfur, and the relevance of glutathione (transport metabolite for reduced sulfur) metabolism during embryogenesis has been highlighted in previous transcriptomic analysis of *P. pinaster* embryogenesis^21^ and functional studies in other species^56^. In addition to its importance in situations of high protein turnover, a link between sulfur metabolism and the synthesis and steady levels of ABA, a major regulator of embryo maturation, has been described in *Arabidopsis*^57^. Thus, it is not surprising to find enrichment of sulfur metabolism processes in our data, given the requirement of ABA across embryo development. In fact, ABA is an essential component of protocols for promoting SE development in conifers^58^.

## Material and Methods

### Plant material

All biological samples have been derived from open-pollinated *P. pinaster* Ait. trees of clone 49, which is part of the Portuguese breeding program population^59^, located in a clonal orchard at Escaroupim National Forest, Portugal (longitude 8°44’W, latitude 39°4’N). The biological samples included zygotic embryos (ZEs), megagametophytes (MGs) and somatic embryos (SEs) (Fig. 1 and Supplementary Table S8). Collection of cones for ZE isolation occurred between mid June and end of July 2012 to 2014. ZEs were isolated as previously reported^21^, and categorized according to the staging system described in Gonçalves *et al*.^60^. Five different groups of developing embryos were considered, as follows: ZE0 included the early embryo stages T0, T1 and T2; ZE3 included the pre-cotyledonary embryo stages T3 and T4; ZE4B included the early cotyledonary embryo stage T4B; ZE5 included the cotyledonary embryo stage T5; and ZE7 included the mature embryo stage T7. Several biological replicates containing between 20–60 embryos were prepared for each of the five groups. The MGs surrounding ZE0 (MG0), ZE4B (MG4B) and ZE7 (MG7) embryos were also isolated and collected in pools of 10 MGs per biological replicate. The SE biological samples were isolated in 2014 from the embryogenic line 49/34/11 (derived from immature embryo tissues of clone 49) submitted to a previously described SE maturation protocol^61^, upon morphological evaluation based on the staging system for ZE^60^. Sixteen early cotyledonary SEs, equivalent to zygotic counterparts ZE4B, were pooled together in SE4B biological sample. Seven late SEs equivalent to ZE7, with a minimum of four cotyledons, were pooled together in SE7 biological sample. All samples were immediately frozen in liquid nitrogen upon collection and stored at −70 °C.

### Equipment and settings

Stereomicroscope observations were performed with a Nikon SMZ800 and images were captured using an Olympus SC30 camera and software.

### RNA isolation and small RNA-Seq

Total RNA samples were extracted using the “Plant/Fungi Total RNA Purification Kit” (NORGEN BIOTEK CORP.), according to manufacturer instructions and with minor modifications: (1) between 600–1000 uL of Lysis Buffer C were added to the biological sample, depending on its complexity; (2) after incubation at 55 °C, the lysate was vortexed twice for 3 min at maximum speed and the clear lysate transferred into a new Eppendorf; (3) total RNA samples were eluted using water. DNA contamination was eliminated using DNase TURBO (Ambion). Cleaned RNA samples were quantified using the QuBit® 3.0 fluorometer and sent to the sequencing service provider who prepared and sequenced the sRNA libraries using Illumina technology.

Two biological replicates were sequenced per tissue/stage, with exception of ZE0, MG0, and SE, for which one biological sample was sequenced.

### MiRNAs identification and annotation

The raw sequencing data files were automatically processed by the sRNA analysis pipeline miRPursuit^62^, with the user-defined criteria described as follows: after a standard pre-processing step, reads were firstly filtered excluding those outside the 18–26 nt range and with an absolute abundance lower than 5; secondly, only reads perfectly mapping (0 mismatches) in the genome of *Pinus taeda* v1.01-masktrim^63^, defined as the Genome^+^DB, were considered to be candidate reads in following steps. Conserved miRNAs were identified/annotated by comparison with mature miRNAs deposited in miRBase v.21, allowing up to two mismatches^17^. Non-conserved reads and conserved miRNAs were processed in order to identify (1) putative novel miRNAs and (2) precursor sequences for all annotated miRNAs, including miRNA-star sequences whenever present in the sRNA library.

### MiRNAs expression analysis

The expression values of annotated miRNAs were normalized against the total number of reads in each library and multiplied by a factor of 10^6^ (CPM or Counts Per Million).

A Principal Component Analysis (PCA) was performed using the “prcomp” R function with the annotated conserved and novel miRNAs and their expression levels after ln(CPM) transformation. The principal components were calculated through correlation matrix, which uses normalized data.

Heatmaps of the conserved miRNA families were built using pheatmap in R after ln(CPM) transformation.

Differentially expressed (DE) miRNAs were determined in ZE samples with an A-NOVA statistical analysis (p-value = 0.05 without alpha correction; assuming ZE0 as an out-group) and in MGs samples with a t-test statistical analysis (p-value = 0.05 without alpha correction, assuming MG0 as an out-group).

### MiRNA target prediction

The targets of the DE miRNAs were predicted using the freely available online tool psRNAtarget^64,65^, against the reference transcriptome *P. pinaster*^66^, and the predicted targets with expectation 3-to-5 were selected for further analysis.

The putative target transcripts of DE miRNAs were subject to a gene enrichment analysis performed with BiNGO plugin from Cytoscape^67,68^, and with the following settings: the Hypergeometric Test as statistical test; the Benjamini & Hochberg’s FDR correction as multiple testing correction; a significance level of 0.05; and whole annotation as the reference set. The Gene Ontology (GO) terms were further summarized by REViGO to remove the redundant ones, using the default options^69^.

### Validation by RT-qPCR

Six conserved miRNAs were selected for expression profile validation based on their high expression levels in ZE samples and on the availability of specific ‘ready to order’ TaqMan miRNA Assays (Applied Biosystems®), namely miR159 (TTGGATTGAAGGGAGCTCCA; Assay ID: 008363_mat), miR162 (TCGATAAACCTCTGCATCCAG; Assay ID: 000342), miR167 (TGAAGCTGCCAGCATGATCTG; Assay ID: 003037_mat), miR171 (TTGAGCCGCGCCAATATCACT; Assay ID: 005375_mat), miR319 (TTGGACTGAAGGGAGCTCCC; Assay ID: 000361), and miR390 (AAGCTCAGGAGGGATAGCGCC; Assay ID: 001409). An absolute quantification of miRNA expression was performed. To this end, oligonucleotides identical to the six selected miRNAs were ordered (Biomers.net), and used to prepare standard curves for each of the selected miRNAs. The cDNA synthesis was performed from 10 ng of DNase-treated total RNA using the TaqMan® MicroRNA Reverse Transcription Kit (Applied Biosystems®) and the miRNA-specific RT primer provided with the TaqMan® MicroRNA Assay (Applied Biosystems®), according to the manufacturer’s instructions. All qPCR experiments were performed in a LightCycler 480 (Roche Diagnostics) with 96-well white plates (Roche Diagnostics). Each 20 uL qPCR reaction mixture included 1X TaqMan® Universal PCR Master Mix II, No UNG (Applied Biosystems®), 1X TaqMan® MicroRNA Assay (Applied Biosystems®) and the cDNA, prepared according to manufacturer’s instructions. Three biological replicates, each with three technical replicates, were used for miRNA expression quantification in ZE and MG samples. For comparative analysis, the small RNA-Seq mean counts of each miRNA in the different biological replicates, and the mean RT-qPCR quantification value were considered.

## Supplementary information

Supplementary Figures

Supplementary Tables (datasets)

## Data Availability

The data were deposited in the European Nucleotide Archive (ENA) under the study PRJEB27796, with the run accessions ERS2608100 to ERS2608104 (MG), ERS2608105 to ERS2608113 (ZE), ERS2616519 to ERS2616520 (SE) [http://www.ebi.ac.uk/ena/data/view/PRJEB27796].
