# Supplementary material for: Small RNA profiling in Pinus pinaster reveals the transcriptome of developing seeds and highlights differences between zygotic and somatic embryos
Source: Sci Rep. 2019 Aug 5;9:11327. doi: 10.1038/s41598-019-47789-y (PMC6683148; doi:10.1038/s41598-019-47789-y)
Supplement: Supplementary file 1 — Supplementary Figures [file 41598_2019_47789_MOESM1_ESM.pdf]

Small RNA profiling in *Pinus pinaster* reveals the transcriptome of developing seeds and highlights differences between zygotic and somatic embryos

Andreia S. Rodrigues[1,2], Inês Chaves[1,2], Bruno Vasques Costa[1,2,3], Yao-Cheng Lin[4,5], Susana Lopes[1,2], Ana Milhinhos[1,2], Yves Van de Peer[5,6,7], Célia M. Miguel[1,2,8]*

[1]iBET, Instituto de Biologia Experimental e Tecnológica, Apartado 12, 2781-901 Oeiras, Portugal

[2]Instituto de Tecnologia Química e Biológica António Xavier, Universidade Nova de Lisboa, Av. República, 2780-157 Oeiras, Portugal

[3]INESC-ID, Instituto Superior Técnico, Universidade de Lisboa, Rua Alves Redol, 9, Lisboa 1000-029, Portugal

[4]Biotechnology Center in Southern Taiwan and Agricultural Biotechnology Research Center, Academia Sinica, Tainan, Taiwan

[5]VIB-UGent Center for Plant Systems Biology, Ghent, Belgium

[6]Department of Plant Biotechnology and Bioinformatics, Ghent University, Ghent, Belgium

[7]Department of Biochemistry, Genetics and Microbiology, University of Pretoria, Private bag X20, Pretoria 0028, South Africa

[8]BioISI – Biosystems & Integrative Sciences Institute, Faculdade de Ciências, Universidade de Lisboa, Lisboa, Portugal

* Correspondence:
Célia M. Miguel
cmmiguel@fc.ul.pt

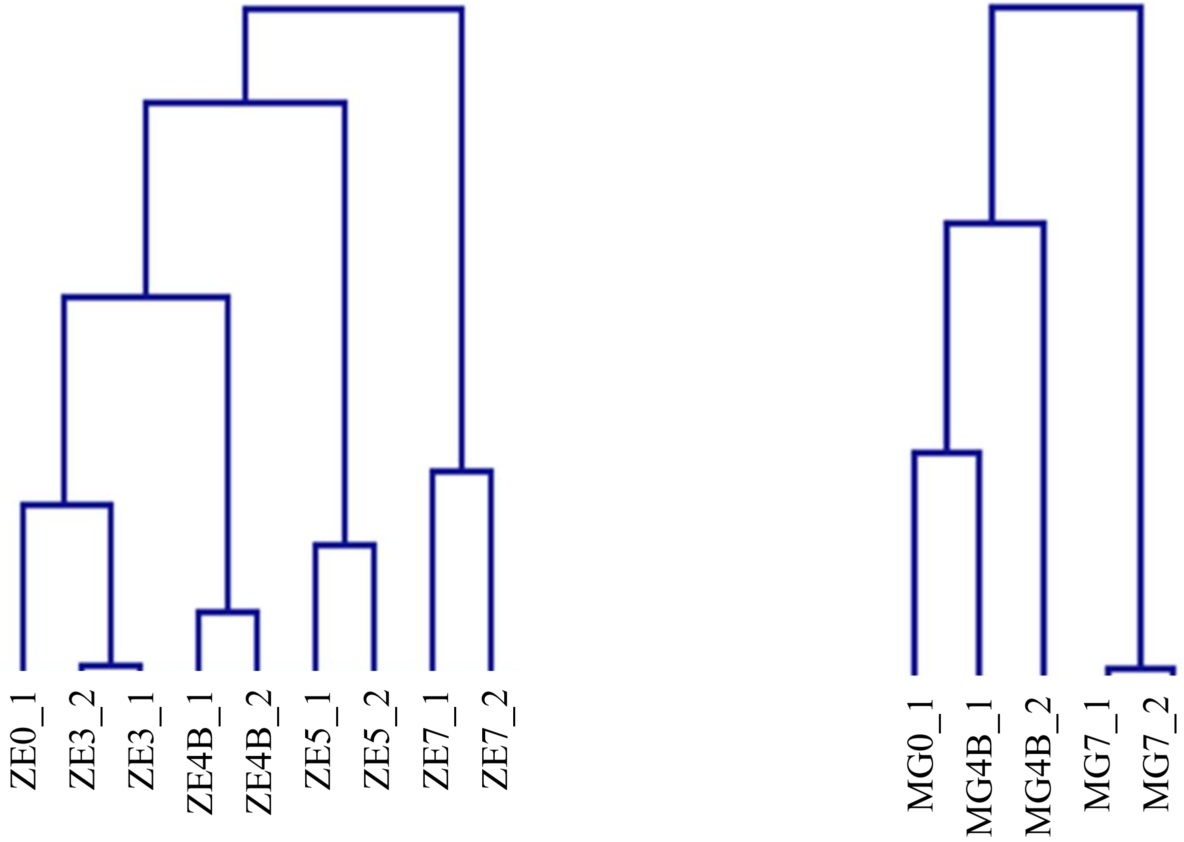

Supplementary Fig. S1 - Hierarchical cluster analysis of ZE and MG samples, using the ln(CPM) for each miRNA and a Kendall's Tau correlation.

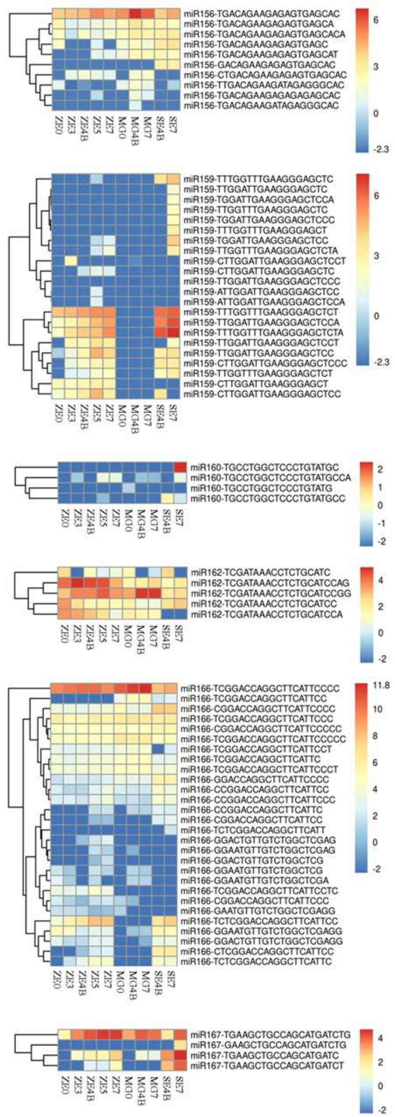

Supplementary Fig. S2 - Conserved miRNA isoforms detected across the distinct developmental stages within each tissue (ZE, MG and SE). The heatmaps represent the transformed expression values for each miRNA isoform, where miRNAs are grouped according to miRNA family and expression profiles across the biological samples.

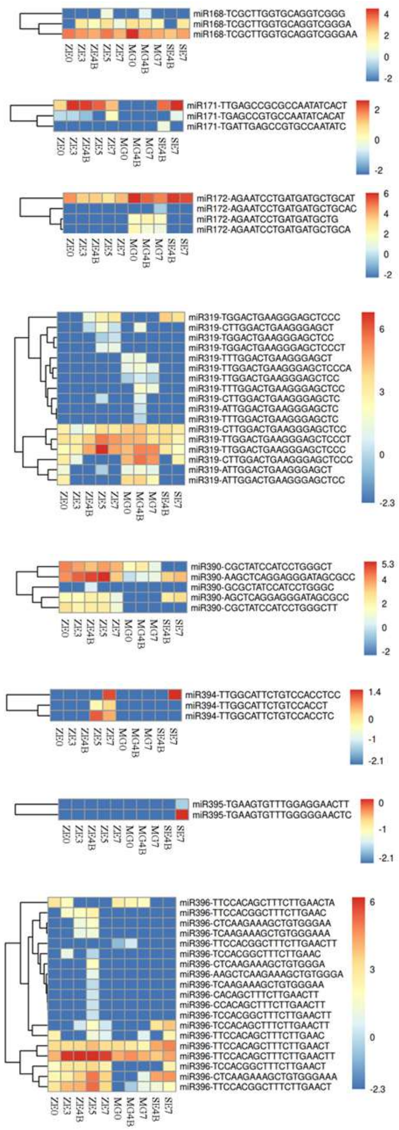

Supplementary Fig. S2 (cont.) - Conserved miRNA isoforms detected across the distinct developmental stages within each tissue (ZE, MG and SE). The heatmaps represent the transformed expression values for each miRNA isoform, where miRNAs are grouped according to miRNA family and expression profiles across the biological samples.

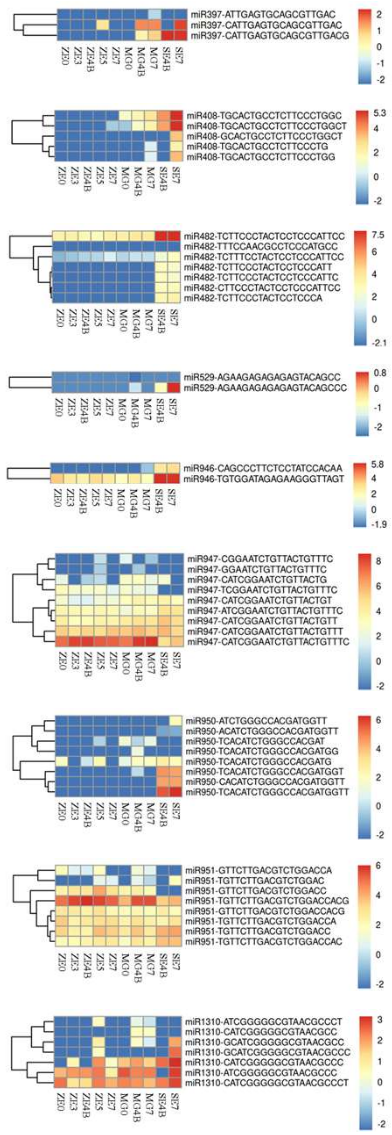

Supplementary Fig. S2 (cont.) - Conserved miRNA isoforms detected across the distinct developmental stages within each tissue (ZE, MG and SE). The heatmaps represent the transformed expression values for each miRNA isoform, where miRNAs are grouped according to miRNA family and expression profiles across the biological samples.

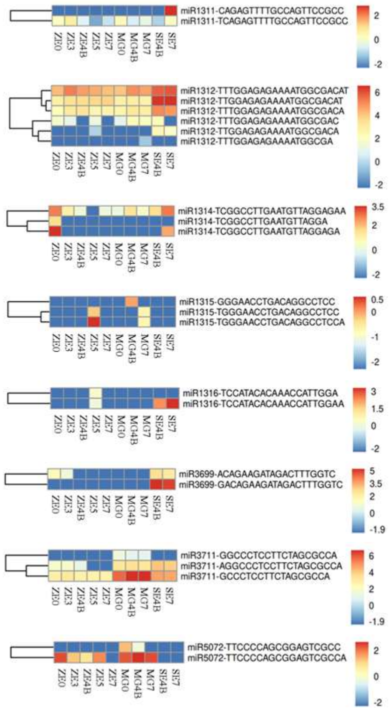

Supplementary Fig. S2 (cont.) - Conserved miRNA isoforms detected across the distinct developmental stages within each tissue (ZE, MG and SE). The heatmaps represent the transformed expression values for each miRNA isoform, where miRNAs are grouped according to miRNA family and expression profiles across the biological samples.

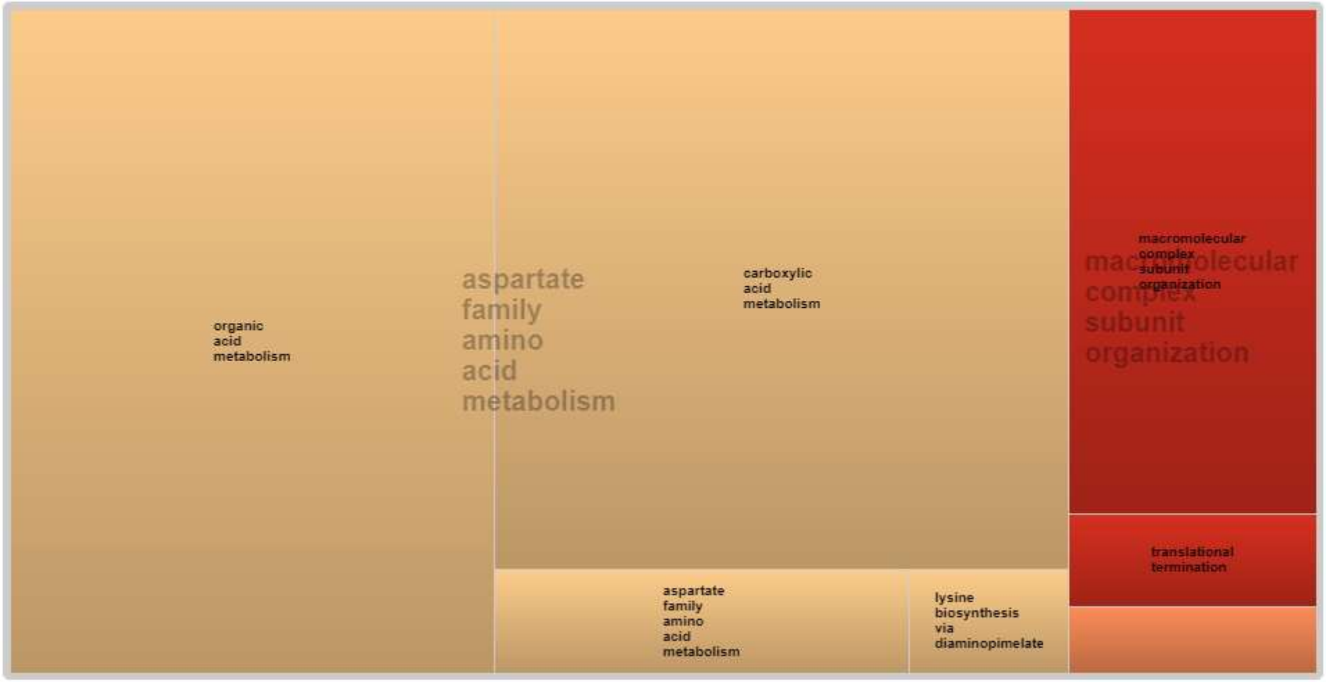

Supplementary Fig. S3 - REViGO TreeMap representation of the BP GO terms found enriched among the miRNAs' targets. The result refers to targets of the differentially expressed miRNAs that follow an expression profile along ZE development included in cluster 2.

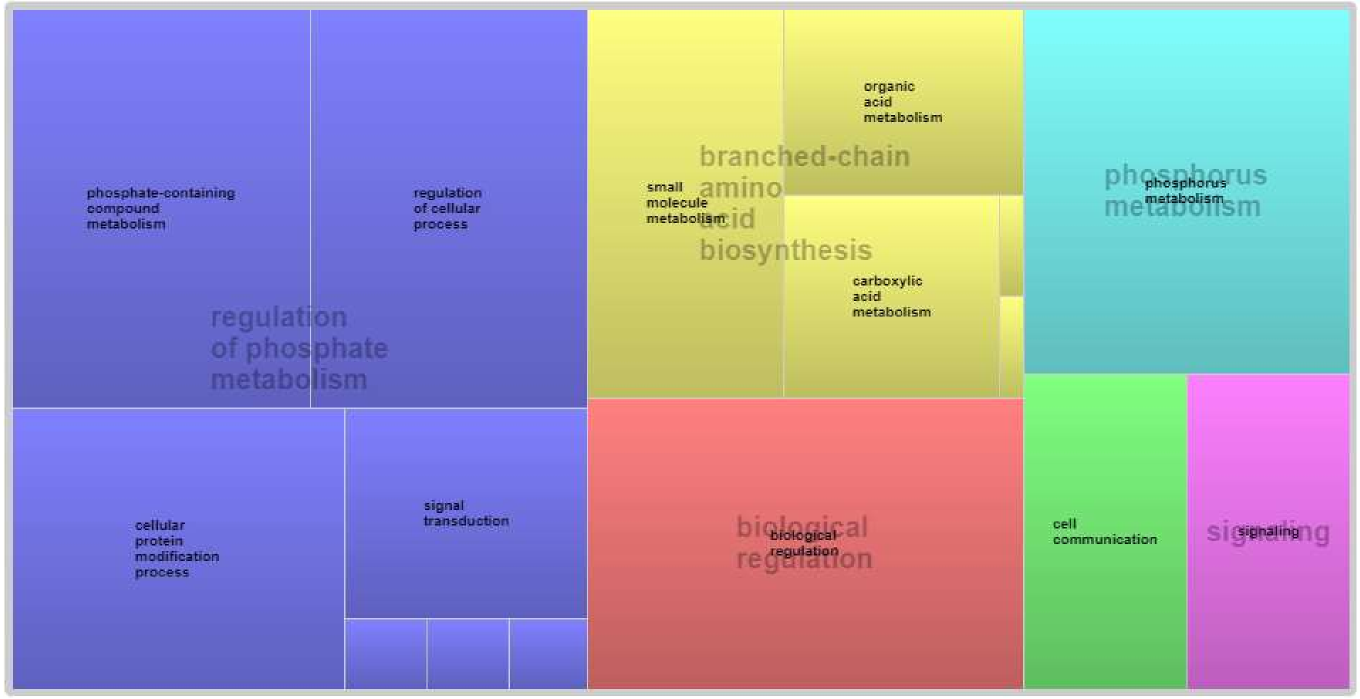

Supplementary Fig. S4 - REViGO TreeMap representation of the BP GO terms found enriched among the miRNAs' targets. The result refers to targets of the differentially expressed miRNAs that follow an expression profile along ZE development included in cluster 4.

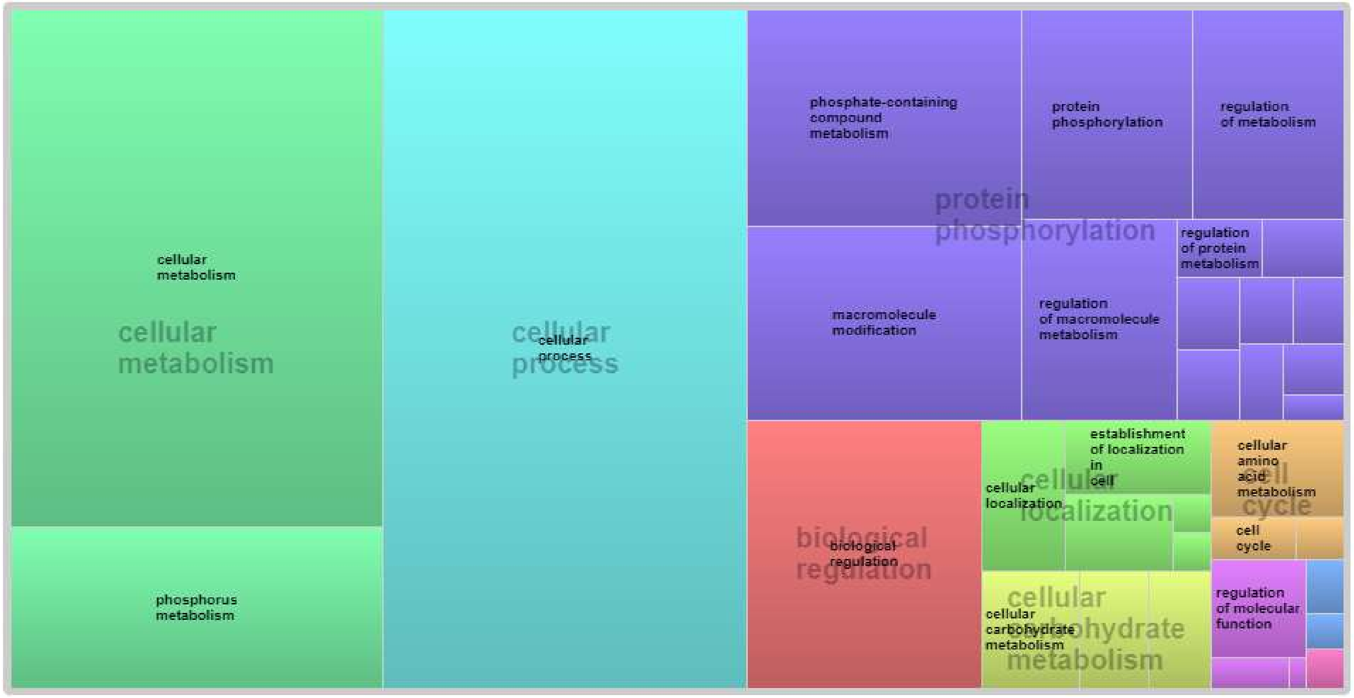

Supplementary Fig. S5 - REViGO TreeMap representation of the BP GO terms found enriched among the miRNAs' targets. The result refers to targets of the differentially expressed miRNAs that follow an expression profile along ZE development included in cluster 6.

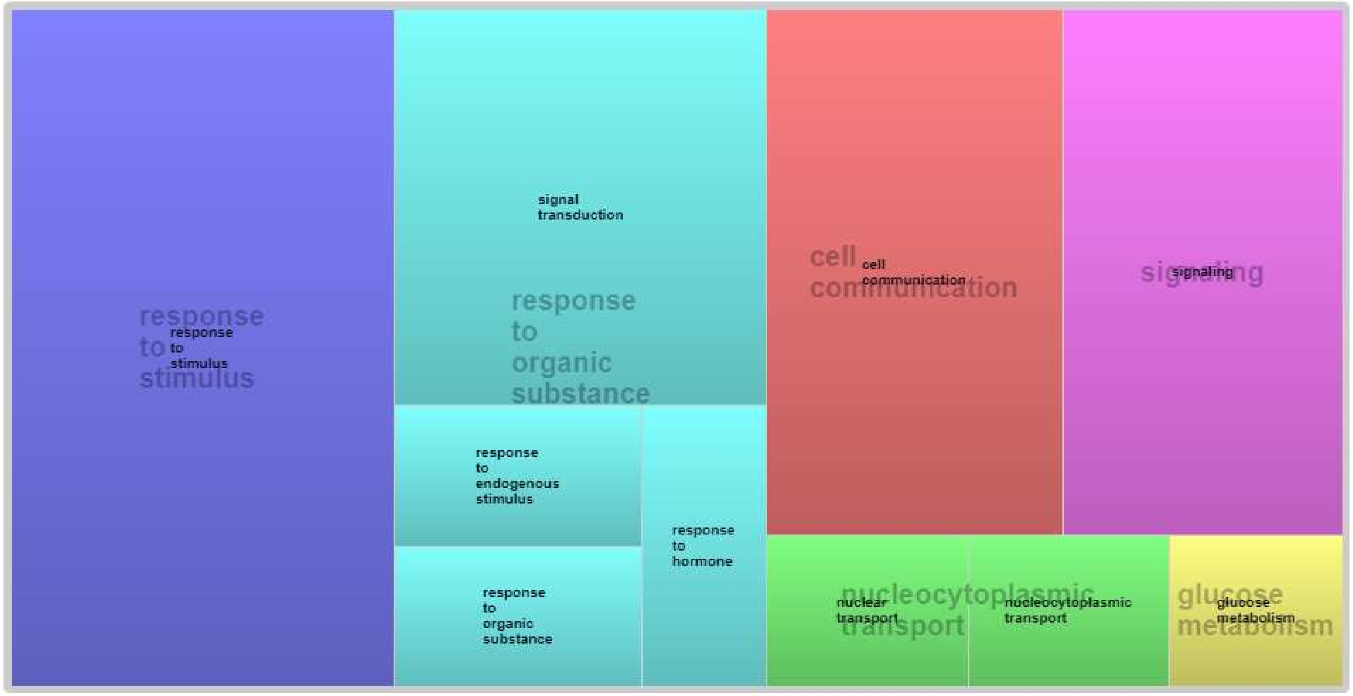

Supplementary Fig. S6. REViGO TreeMap representation of the BP GO terms found enriched among the miRNAs' targets. The result refers to targets of the differentially expressed miRNAs that follow an expression profile along MG development included in cluster 2.
